# The Effects of Consuming White Button Mushroom *Agaricus bisporus* on the Brain and Liver Metabolome Using a Targeted Metabolomic Analysis

**DOI:** 10.3390/metabo11110779

**Published:** 2021-11-15

**Authors:** Gloria I. Solano-Aguilar, Sukla Lakshman, Saebyeol Jang, Richi Gupta, Aleksey Molokin, Steven G. Schroeder, Patrick M. Gillevet, Joseph F. Urban

**Affiliations:** 1 Diet Genomics and Immunology Laboratory, Beltsville Human Nutrition Research Center, Agricultural Research Service, U.S. Department of Agriculture Northeast Area, Beltsville, MD 20705, USA; Sukla.Lakshman@usda.gov (S.L.); Saebyeol.Jang@gmail.com (S.J.); Aleksey.Molokin@usda.gov (A.M.); Joe.Urban@usda.gov (J.F.U.J.); 2 Microbiome Analysis Center, George Mason University, Science & Technology Campus, Manassas, VA 20108, USA; indorichi@gmail.com (R.G.); pgilleve@gmu.edu (P.M.G.); 3 Animal Genomics and Improvement Laboratory, Beltsville Agricultural Research Center, Agricultural Research Service, U.S. Department of Agriculture Northeast Area, Beltsville, MD 20705, USA; Steven.Schroeder@usda.gov

**Keywords:** white button mushroom, targeted metabolomics, hippocampus, cortex, liver, glycerophospholipids, sphingomyelin, amino acids

## Abstract

A targeted metabolomic analysis was performed on tissues derived from pigs fed diets supplemented with white button mushrooms (WBM) to determine the effect on the liver and brain metabolome. Thirty-one pigs were fed a grower diet alone or supplemented with either three or six servings of freeze-dried WBM for six weeks. Tissue metabolomes were analyzed using targeted liquid chromatography-mass spectrometry (LC-MS) combined with chemical similarity enrichment analysis (ChemRICH) and correlated to WBM-induced changes in fecal microbiome composition. Results indicated that WBM can differentially modulate metabolites in liver, brain cortex and hippocampus of healthy pigs. Within the glycero-phospholipids, there was an increase in alkyl-acyl-phosphatidyl-cholines (PC-O 40:3) in the hippocampus of pigs fed six servings of WBM. A broader change in glycerophospholipids and sphingolipids was detected in the liver with a reduction in several lipid species in pigs fed both WBM diets but with an increase in amino acids known as precursors of neurotransmitters in the cortex of pigs fed six servings of WBM. Metabolomic changes were positively correlated with increased abundance of *Cryomorphaceae*, *Lachnospiraceae*, *Flammeovirgaceae* and *Ruminococcaceae* in the microbiome suggesting that WBM can also positively impact tissue metabolite composition.

## 1. Introduction

Edible mushrooms are consumed as a delicacy for their flavor and nutritional value derived from high quality protein, essential amino acids, fiber, and a low-fat content [1,2]. Carbohydrates including structural polysaccharides like β-glucan, chitin, hemicellulose and pectin constitute the most abundant macronutrient of mushrooms, followed by protein and unsaturated fatty acids with a higher contribution of linoleic acid; an important precursor of long-chain n-6 fatty acids for humans [1,3]. Edible mushroom derived proteins lectins [2], amino acid ergothioneine [4], flavonoids [5] and polysaccharides categorized as glucans with β-type glycosidic bonds have been proposed as bioactive components responsible for regulation of the gut microbiome, modulation of the host immune response, hypolipidemic and anti-oxidant activity [6,7,8,9,10,11]. In addition, neuroprotective and anti-ageing properties of mushroom and isolated compounds like ergothioneine have been proposed for use against age related disorders [12,13,14,15,16,17,18], through the anti-inflammatory action purportedly regulated by changes in the composition of the intestinal microbiome. However, more preclinical evidence is required to understand the potential mechanism of action of dietary mushroom in treatment or neurological disease prevention.

The brain is highly enriched in lipids including cholesterol, phosphatidylcholine, sphingomyelin, ceramides, glucosyl-ceramides and sulfatides that are essential for central nervous system functions such as neurotransmission, synaptic plasticity, and nerve impulse conduction [19]. Gut bacterial communities are dynamic entities that change in composition and function in response to environmental factors such as diet and can influence the bidirectional communication of the microbiome–gut–brain axis through various pathways including immune system, neuroendocrine responses and bacteria-derived metabolites [20,21]. Several neurobiological mechanisms have linked the consumption of unhealthy diets such as westernized-diet (WD) or diets rich in saturated fats with alterations in the gut microbiome that potentially contribute to marked differences in the brain lipidome and cognitive dysfunction [22,23,24]; however, research into the potential benefits of healthy diets in modulating these microbiome–brain interactions are only starting to emerge [23]. A comprehensive assessment of the impact of diet on brain health through metabolomics analysis recently suggested a protective association between metabolites derived from polyphenol rich foods and reversal of cognitive decline [18]. There also appears to be a role for microbiome-derived metabolites in gut–brain communication and regulation of brain function [25].

We have previously shown that feeding WBM to healthy pigs induced a prebiotic effect that influenced the composition of the gut microbiome with enrichment of beneficial bacteria associated with carbohydrate metabolism and short chain fatty acid (SCFA) production and a reduced inflammatory response detected in cultured macrophages derived from WBM-treated pigs after lipopolysaccharide (LPS) stimulation [26]. Here, we extended our initial observations to assess the effect of WBM dietary supplementation on the brain and liver metabolome and its relationship with WBM-induced changes in the intestinal microbiome.

## 2. Results

### 2.1. Metabolome Profile Analysis

**Figure 1:**
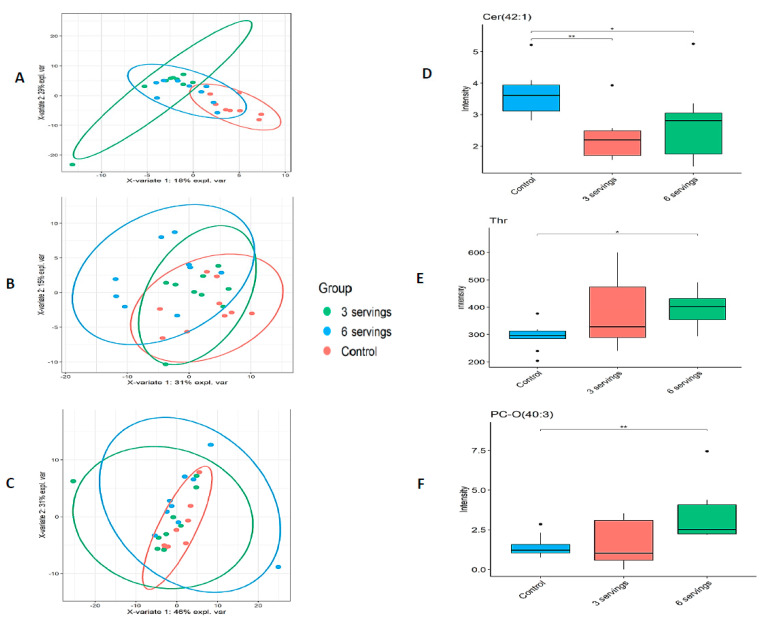
Partial least squares discriminant analyses (PLS-DA) among treatment groups in the liver and brain of pigs fed WBM-supplemented or control diets. PLS-DA plots showed separation of metabolites in the liver of pigs fed three servings of WBM-supplemented diet (**A**), the cortex of pigs fed six servings of WBM (**B**) and no significant separation in the hippocampus regardless of feeding WBM (**C**) (*p* < 0.05) compared to pigs fed a control diet. Boxplot of top representative metabolite in liver: ceramide (Cer 42:1) (**D**), cortex: threonine (Thr) (**E**), and hippocampus: 1-alkyl,2-acylglycero-3-phosphocholine PC-O (40:3) (**F**) showing significant change in metabolite abundance after dietary treatment, * denotes *p*-value < 0.05, ** *p*-value < 0.01.

Out of the 408 metabolites included in the kit, 154 (38%), 158 (39%) and 167 (41%) metabolites were detected across eleven different classes above the limit of detection from pre-frontal cortex, hippocampus, and liver, respectively (Table 1). Partial least squares discriminant analysis in all tissues indicated a separation of liver metabolites derived from pigs fed three servings of WBM (AUC = 0.84, *p* < 0.01) and pigs fed the control diet (AUC = 0.97, *p* < 0.01) (Figure 1A); a partial separation of cortex metabolites derived from pigs fed six servings of WBM (AUC = 0.78, *p* = 0.02) (Figure 1B), and no separation of hippocampus metabolites among the three treatment groups (Figure 1C) with top representative metabolite in liver: ceramide (Cer 42:1) (Figure 1D), cortex: threonine (Thr) (Figure 1E), and hippocampus: 1-alkyl,2-acylglycero-3-phosphocholine PC-O (40:3) (Figure 1F) showing significant change in metabolite abundance after dietary treatment.

As all metabolites did not meet the assumption of a normal distribution, the Mann–Whitney U test was performed to identify differentially expressed metabolites in every organ among treatment groups. Fold change (FC) value for each comparison were calculated and used to create comparative heatmaps among treatment groups (Figure S1). Differences in mean rank among treatment groups with calculated FC and variable importance projection score (vip) for each metabolite are summarized in Table 2, Table 3 and Table 4. In medial pre-frontal-cortex, there was a 1.3 FC increase in essential amino acid threonine and 1.2 FC increase in diglyceride DG (42:2) (FDR < 0.05) with a 1.2 FC increase in glutamine and essential amino acid methionine (FDR < 0.1) with a vip score > 1.4 in pigs fed six-servings of the WBM-supplemented diet. The abundance of several glycerophospholipid and sphingolipid metabolites in the group fed six-servings of WBM showed a non-significant increase relative to the control fed group with a vip score > 1.4 contributing to the separation among other treatment groups (Table 2).

In hippocampus, there was a 2.1 FC increase in alkyl-acyl-phosphatidylcholine PC-O (40:3) (FDR < 0.05) in pigs fed the six serving of WBM-supplemented diet relative to control group. Within phosphatidylcholine class, the abundance of PC (39:5) and PC (42:6) was decreased in pigs fed six- and three servings of WBM relative to pigs from the control group, but with a lower vip score (vip < 1.4) (Table 3). As in the cortex, changes in the abundance of other glycerophospholipid and sphingolipid metabolites in the hippocampus of pig fed six-servings of WBM showed a non-significant increase relative to the control group with a vip score > 1.4. A broader lipid metabolite profile was detected in liver samples with all differentially expressed metabolites showing a vip > 1.4. Lipidomic analyses revealed a reduction in sphingolipids: sphingomyelins SM (42:1), SM (43:1) and ceramide (42:1) in pigs fed both WBM-supplemented diets relative to control group (FDR < 0.05) with a significant two-fold increase in serotonin in pigs fed three servings of WBM (FDR < 0.05). Within the phosphatidylcholines, there was a reduction in abundance of PC (31:1), (35:4), (37:1), (46:2) and PC-0 (35:4) in pigs fed both WBM-supplemented diets and PC (33:1), (35:1), (39:3), PC-0 (33:4) in those pigs fed three servings of WBM relative to controls. Lyso-alkyl-phosphatidylcholine LPC (15:0) was also reduced in pigs fed both WBM-supplemented diets relative to control group (FDR < 0.05). Only PC (38:2) and (32:6) were increased in liver from pigs fed both WBM-supplemented diet groups (Table 4).

### 2.2. Metabolic Analysis by ChemRICH

**Figure 2:**
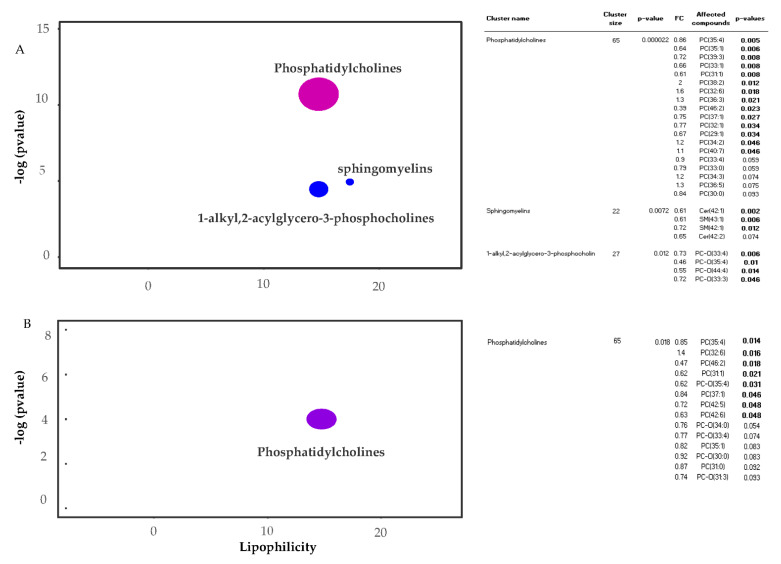
ChemRICH analysis plot for liver from pigs fed three or six serving of WBM. The most significant altered families of metabolites are shown on the *Y*-axis. Enrichment *p*-values are given by Kolmogorov–Smirnov test and displayed along the ordinate, with *p*-values transformed in −log10. Disc sizes represent the total number of metabolites in each group set. Red discs represent increased metabolites while blue represent decreased metabolites in pigs fed WBM-supplemented versus control diets. Intermediate purple color represents the presence of both increased and decreased metabolites. ChemRICH impact plot for three servings of WBM vs. control fed pigs (**A**) and for six servings of WBM vs. control fed pigs (**B**) with corresponding statistic table for each metabolite cluster. *p*-values < 0.05 in bold.

**Figure 3:**
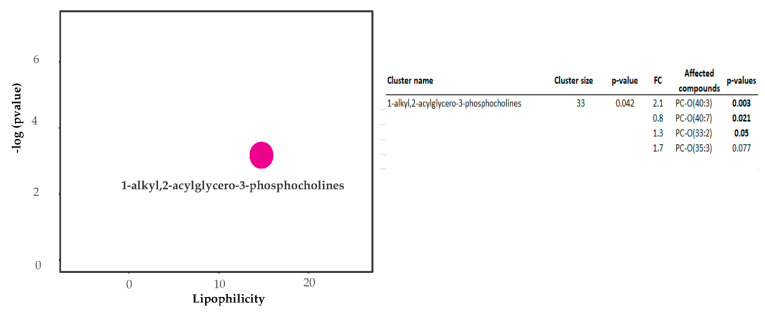
ChemRICH analysis plot for hippocampus from pigs fed six serving of WBM. ChemRICH impact plot for pigs fed six servings of WBM vs. pigs fed control diet with corresponding statistic table showing affected compounds within the alkyl,2-acylglycero-3-phosphocholines. *p*-values < 0.05 in bold.

ChemRICH analyses were applied to group metabolite responses in each organ based on structure similarities and chemical ontology. The ChemRICH database uses chemical similarity to group metabolites that are significantly altered within clusters. There was a general decrease in the quantity of phosphatidylcholines, 1-alkyl,2-acylglycero-3-phosphocholines and sphingomyelins in the livers of pigs fed three serving of WBM and only phosphatidylcholines in those fed six servings of WBM (Figure 2). Within the brain, there was predominantly an increase in the abundance of 1-alkyl,2-acylglycero-3-phosphocholines in the hippocampus of pigs fed the six serving of WBM (Figure 3) with no detectable difference within metabolite clusters by ChemRICH in the cortex.

### 2.3. Associations of Metabolites with Fecal Microbiota

**Figure 4:**
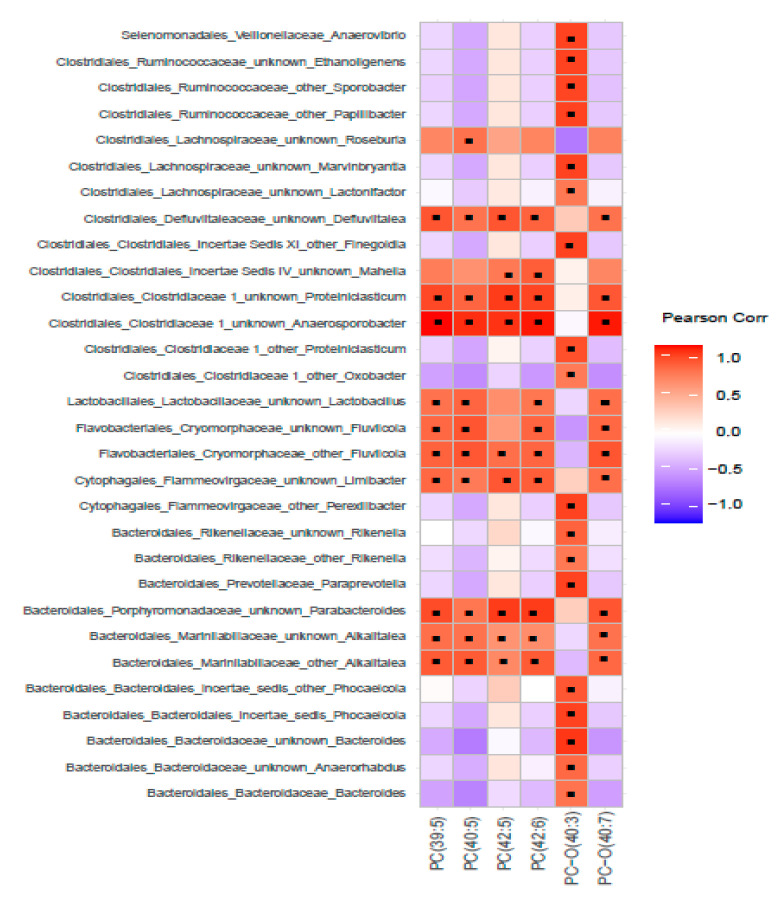
Correlation between the composition of bacteria in the fecal microbiome and alkyl-acyl-glycerol phosphocholines in the hippocampus of pigs fed six servings of WBM. Pearson Correlation coefficients were estimated for each pairwise comparison of bacterial taxa and metabolites within the species of alkyl-acyl-glycerol phosphocholine lipids. Black dots within heatmap represent significant relationships (*p*-value < 0.05).

To understand the relationship between fecal microbiota (FM) derived from pigs fed six servings of WBM and tissue metabolites, correlation heatmaps and interaction network analysis were constructed at the end of a six-week feeding regimen and compared among treatment groups to visualize change in relationships after dietary intervention. Within glycerophospholipids in the hippocampus, there were several positive correlations between alkyl-phosphatidylcholine (PC-O 40:3), the top identifiable metabolite by univariate and ChemRICH analysis, and genus from *Bacteroidaceae*, *Rikenellaceae, Prevotellaceae* families within Bacteroidales and *Clostridiaceae*, *Lachnospiraceae* and *Ruminococcaeae* families within Clostridiales. In addition, PC-0 (40:7) and phosphatidylcholines PC (39:5), (40:5), (42:5) and (42:6) were all positively correlated with genus within *Marinilabilaceae* (Alkalitalea), *Porphyromonadaceae* (Parabacteroides), *Cryomorphaceae* (Fluviicola) and genus within *Clostridicaeae* and *Defluviitaleceae* families (Figure 4) which have previously been identified as responsive taxa to WBM dietary supplementation [26]. Some of the same genus within *Marinilabilaceae* (Alkalitalea), *Porphyromonadaceae* (Proteiniphilum), *Cryomorphaceae* (Fluviicola) families in addition to *Flammeovirgaceae* (Limibacter) were also positively correlated with several alkyl-phosphatidylcholine and some lyso-phosphatidylcholine in cortex but no clear pattern found with PC or amino acids (Figure S2). In liver, there was a negative correlation between sphingomyelin and bacteria within *Porphyromonadaceae*, *Lachnospiraceae* and *Ruminococcaceae* families with not a defined relationship for the limited group of glycerophospholipids (Figure S3).

**Figure 5:**
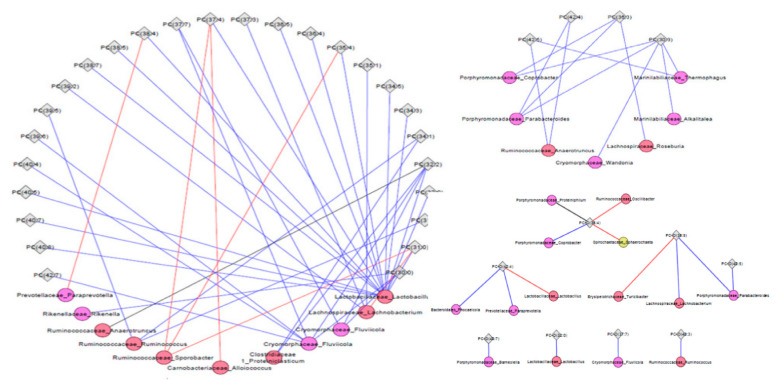
Bacteria-metabolite network visualization in the hippocampus of pigs fed six servings of WBM-supplemented diet. Bacterial genus in the fecal microbiome and the most abundant metabolite lipid species phosphatidylcholine and alkyl-acyl-glycerol phosphocholines compared between pigs fed six servings of WBM versus and control diets. Bacterial genus is represented by circles and hippocampus metabolites by diamonds. Change from negative to positive correlation are shown in blue, while change from positive to negative is shown in red. Only significant correlations are shown (*p*-value < 0.05).

**Figure 6:**
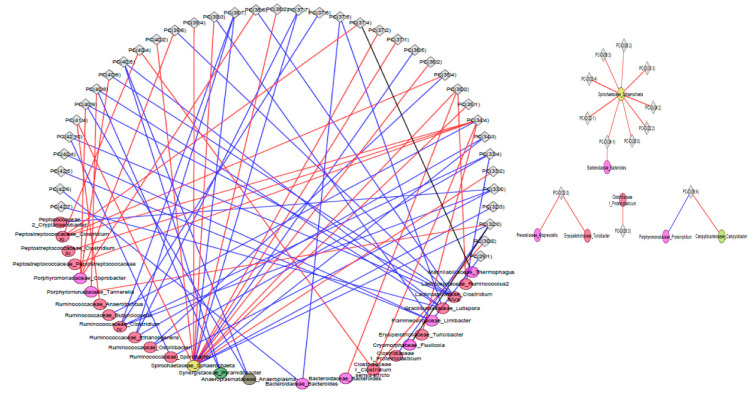
Bacteria-metabolite network visualization in the cortex of pigs fed six servings of a WBM-supplemented diet. Bacterial genus in the fecal microbiome and the most abundant metabolite lipid species phosphatidylcholine and alkyl-acyl-glycerol phosphocholines were compared between pigs fed six servings of WBM-supplemented versus control diets. Bacterial genus is represented by circles, cortex metabolites are represented by diamonds. Change from negative to positive correlation are shown in blue, while change from positive to negative are shown in red. Only significant correlations are shown (*p*-value < 0.05).

**Figure 7:**
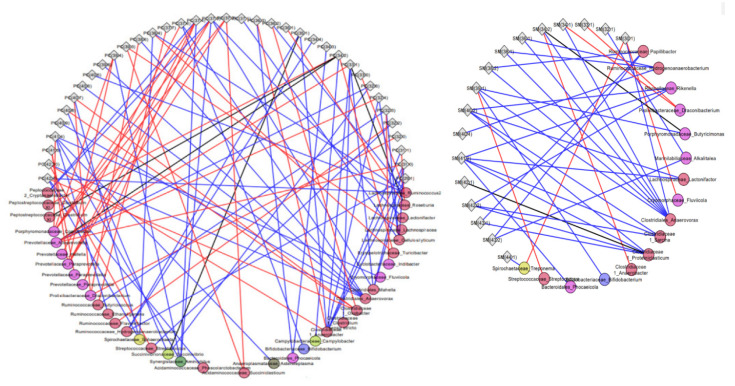
Bacteria-metabolite network visualization in the liver of pigs fed six servings of a WBM-supplemented diet. Bacterial genus in the fecal microbiome and the most abundant metabolite lipid species phosphatidylcholine and sphingomyelin compared between pigs fed six servings of WBM versus control diets. Bacterial genus is represented by circles, liver metabolites are represented by diamonds. Change from negative to positive correlation are shown in blue, while change from positive to negative in shown in red. Only significant correlations are shown (*p*-value < 0.05).

Correlative changes between relative abundance of gut microbiota and tissue metabolites were also calculated among pigs fed six servings of WBM and control groups after a six-week dietary intervention and the top five metabolite classes with the largest changes were visualized using microbiota-metabolite network plots using Cytoscape (Table S1). Metabolites within phosphatidylcholine (53 out of 67) and alkyl-phosphatidylcholine (13 out of 15) species positively correlated with bacteria predominantly from *Lactobacillaceae, Cryomorphaceae, Porphyromonadacaeae and Ruminococcaceae* families in hippocampus of pigs fed six servings of WBM relative to the control diet group (blue lines) with few negative correlations (red lines) (Figure 5). In cortex, pigs fed the six servings of WBM showed a positive correlation between 43 out of 79 metabolites within phosphatidylcholines (blue lines) and bacteria from *Ruminococcaceae* and *Lachnospiraceae* families and negatively correlated with bacteria from *Porphyromonadaceae, Peptostreptococcaceae* and *Spirochateaceae* families (red lines) (Figure 6). Metabolites within phosphatidylcholines (66 out of 113) and sphingomyelins (38 out of 49) were the top metabolite species in the liver positively correlated with genus predominantly from *Clostridiaceae* and *Bifidobacteriaceae* families that showed a reduced abundance in response to feeding WBM relative to control diet [26]. In contrast, the abundance of *Cryomorphaceae* was negatively correlated with sphingomyelin (Figure 7).

### 2.4. RNA Sequencing of Brain

No DGE were found in hippocampus, cortex or hypothalamus of pigs fed WBM for the 30,476 and 12,189 genes mapped to the swine genome Ensemble build 11.1 98_111 [27] or the non-redundant porcine gene library [28], respectively. A mitochondrial gene *mtATP8* coding for a complex V protein of the mitochondrial respiratory chain [29] was increased in all three brain sections (cortex, hippocampus, hypothalamus). However, RT-PCR validation of *mtATP8* expression indicated that a few pigs fed three- and six servings of WBM were responsible for the non-significant increase (Figure S4).

## 3. Discussion

A targeted metabolomic analysis was used to assess the changes in metabolite profile in the liver and brain of pigs fed a growth diet supplemented with the equivalent of 75 grs or 150 grs of fresh white button mushroom (WBM) daily for six weeks. A LC-MS analysis combined with determination of chemical similarity enrichment showed that consumption of WBM modulated the metabolite composition of the liver and brain cortex and hippocampus of healthy pigs. Within the glycerophospholipids, the major lipid component found in cell membranes, there was a mixed response to feeding WBM with an increase in alkyl-acyl-phosphatidylcholine PC-O (40:3) in hippocampus of pigs fed six serving of WBM and a decreased abundance of phosphatidylcholines PC 39:5 and 42:6 in pigs fed the three- and six servings of WBM compared to pigs fed the control diet. A broader change in glycerophospholipids and sphingolipids was detected in the liver with a reduction in several phosphatidylcholines (PC), alkyl-acyl-phosphatidylcholines (PC-O), lyso-alkyl-phosphatidylcholines (LPC), sphingomyelins (SM) and ceramides (Cer) in both WBM fed groups and increased serotonin in the pigs fed three-servings of WBM relative to controls. In the brain cortex there were no significant changes in glycerophospholipid abundance; however, there was an increase in amino acids known as precursors of neurotransmitters such as glutamine, methionine and threonine known to facilitate healthy brain activity [30]. The regulation of cerebral lipidome homeostasis is not fully understood but it has been shown that it can be partially modulated as a consequence of changes in dietary lipids with increased use of saturated fatty acids [24,31] or deficiency of n-3 fatty acid, which provoked region-specific changes in the fatty acid composition of the brain with cortex being more severely affected [32]. Compared to other foods of vegetable or animal origin, mushroom lipids consist mainly of mono- and polyunsaturated fatty acids, often with a ratio of unsaturated to saturated fatty acids that classifies them as a health source of lipids. WBM contains high level of long chain polyunsaturated fatty acids LC-PUFA, in specific linoleic acid [3] known to be a precursor for essential LC-PUFA particularly arachidonic acid (AA: 20:4 n-6) for vertebrates who are unable to synthesize them [32].After dietary intake, PUFAs are actively incorporated into cells as acyl chains of membrane glycerophospholipids [19] and other lipid classes with an strong impact on lipidome profiles of metabolic organs such as the liver while causing less of an impact on other organs like the brain [33]. While our study of metabolite composition in response to WBM dietary supplementation in healthy pigs was not designed to indicate whether WBM prevents disease, there is support for a beneficial role of eating WBM to promote host health through increased amino acid that serve as precursors for neurotransmitters in the brain cortex [30], increased abundance of alkyl-acyl-glycerol phosphocholines in hippocampus associated with signaling pathways [34] and reduced phosphatidylcholines, lyso-phosphatidylcholine and sphingomyelins in the liver that have been used as disease biomarkers of oxidative stress and inflammation [35]. Changes in brain metabolite composition have been associated with ageing and have been used as a diagnostic feature to differentiate neurodegenerative conditions. Glycerophospholipid, and sphingolipid accumulation in cerebrospinal fluid (CSF) [36] and plasma [37] have shown an increased lipid turnover in subjects with Alzheimer’s disease (AD) consistent with the formation of inflammatory lipids [38,39,40,41,42]. Similar changes in inflammatory lipid species have also been found in Parkinson’s disease [43] and Multiple sclerosis [44], as well as metabolic diseases such as type 2 diabetes [45], non-alcoholic steatohepatitis [46] and non-alcoholic fatty liver [47] where liver biopsies and high serum levels of sphingomyelins have been used as biomarkers of visceral adipose tissue and hepatic triglyceride content [47,48]. A protective association between metabolites reflecting the consumption of mushroom and brain health have been proposed in a prospective cognitive decline study with humans [18] and after oral administration of the mushroom derived antioxidant ergothioneine in a recognition memory study in rodents [17]. This information provides additional evidence for improved mental health through diets that alter the gut microbiome to positively interact with the central nervous system.

Altered bacterial composition of the gut microbiome [49] and extracellular bacterial vesicle [50,51] have been linked to brain impairment and behavioral dysfunction. The microbiome–gut–brain axis plays an important role in regulation of brain function as demonstrated with westernized diet-induced disruption of cognitive function [52], the induction of chronically depressive-like behavior with fecal microbiota transplantation [53], improved learning and memory in mice treated to reverse obesity, and reduced brain function associated with antibiotic alterations in the composition of the gut microbiome [54]. Lipidomic profiling has shown excessive dietary lipid induced cellular stress and lipotoxicity with increases in diacylglycerides, lyso-phosphatidylserines, ceramides, phosphatidylcholines (PCs) in the hippocampus [55] and hypothalamus which is not readily reversed by exercise [56]. Disturbances in the gut microbiome have also been linked to neuroinflammation in a Parkinson disease model with identified decreases in bacteria from *Lachnospiraceae* and increased abundance of the Enterobacteriaceae family [57]. Similarly, disturbed balance of the colonic mucosal microbiome has also been linked to memory impairment in cirrhotic patients [58] suggesting dietary modulation of the microbiome can potentially be used for microbiome-based interventions targeting metabolite linked to neurodegenerative diseases as changes in microbial composition most likely cause changes in metabolite levels [59].

It is well documented that the gut microbiome may positively affect host metabolism through the regulation of dietary metabolites like SCFAs, bile acids and amino acid derivatives whose products can influence neurotransmitter production [60] and cerebral function [61,62]. We previously demonstrated a prebiotic effect of feeding WBM to healthy pigs that affected the composition and function of the fecal microbiome with a reduced inflammatory response to LPS-induced cell activation ex vivo [26]. Here, we present additional evidence that feeding WBM-supplemented diets to pigs for six weeks can also affect the brain and liver metabolome associated with changes in the intestinal microbiome. An increased correlation between acyl-alkyl-phosphatidylcholine in the hippocampus and cortex and bacteria within the *Cryomorphaceae* (Fluviicola), *Flammeovirgaceae* (Limibacter) [26] and within *Marinilabilaceae* (Alkalitalea), an anaerobic haloalkaliphilic bacterium responsible for xylan degradation in wheat-straw based compost used for *Agaricus bisporus* growth [63,64], suggested that this lipid species was significantly increased in the brain by feeding WBM. Interaction network analysis indicated a positive correlation between phosphatidylcholine (PC) in the hippocampus and cortex and bacteria within the *Lactobacilaceae*, *Cryomorphaceae*, *Ruminococcaceae*, *Lachnospiraceae, Flammeovirgaceae* families induced by feeding six servings of WBM diet. Bacteria within the *Lachnospiraceae*, *Ruminococcaceae* and *Eryipelotrichaceae* families have been previously linked with enhanced behavioral performance in a maternal-obese animal model that demonstrated that the gut microbiota differentially affected cognitive function in offspring [65]. Dietary interventions with soluble dietary fiber β-glucan, which is fermented in the lower gastrointestinal tract, have also been associated with improved cognitive function in a diet-induced obesity model suggesting causality between an altered gut microbiota and cognition [66]. Diet-induced increases in bacteria within the Bacteroidales restored β-glucan induced cognitive improvements ablated by antibiotic treatment [66]. In addition, phosphatidylcholine (PC) dietary supplementation has been shown to prevent LPS-induced systemic inflammation and synaptic damage via the gut–brain axis [67] associated with increases in the relative abundance of Lactobacillus and Bifidobacterium, *Rickenellaceae* and *Lachnospiraceae* families [67].

The novelty of our study is the demonstration that changes in the brain metabolome were related to WBM induced changes in the fecal microbiome. The diet effect of feeding WBM on specific regions of the brain may be related to the sensitivity of the brain to circulating metabolites in the serum moving across the blood–brain barrier to alter the brain metabolic profile [24,68]. Lipidomic profiles in the serum can differ from the tissues such as the liver, brain and heart [69]. Even within the same organ, different regions showed distinct lipidomic profiles [69]. Given that feeding WBM can alter the intestinal microbiome producing circulating amino acids and lipids that can potentially alter the production of neurotransmitters in cortex suggests that dietary modulation of the intestinal microbiome can potentially be used for targeting neurodegenerative diseases. Further characterization of diet induced changes in the intestinal microbiome that enhance production of circulating metabolic precursors that alter production of neurotransmitters in the brain should provide strategies to use nutrition to positively impact metabolomic function of the brain.

## 4. Materials and Methods

### 4.1. Ethics Statement

All animal experiments and collection of samples were conducted in accordance with guidelines established and approved by Beltsville Animal Care and Use Committee under protocol no. 13-028.

### 4.2. Diet Formulation and Tissue Sample Collection

Thirty-one commercial Landrace X Duroc crossbred six-week-old pigs were randomized by weight and balanced groups by gender into three experimental treatment groups with isocaloric diets (10–11/group). Group I was fed a grower diet (13% energy (E) from fat, 69% E from carbohydrates and 18% E from protein) that was used as a control group. Group II was fed grower diet supplemented with three servings of freeze-dried WBM equivalent to 75 g of fresh WBM fed to a human weighing 65–70 kgs, and Group III was fed a grower diet supplemented with six servings of freeze-dried WBM equivalent to 150 g of fresh WBM per day for a six-week intervention as previously described [26]. The brain was removed from pigs euthanized using Euthasol^®^ (Virbac AH, Inc., Fort Worth, TX, USA). The location of hypothalamus, hippocampus and medial pre-frontal cortex were identified using a pig atlas [70] and following previously described anatomical markers [71]. The location of the hypothalamus was ventro-rostral to the thalamic region, dorsal to the optic chiasm, and ventral to the section of the fornix that was visible. It also makes up a part of the rostral wall of the third ventricle. The hippocampus located in the temporal horn of the lateral ventricle and localized ventral to the corpus callosum. To remove the medial pre-frontal cortex, tissue was dissected from the frontal gyrus to the corpus callosum, removing the most medial portion of that section leaving the right cortex [71]. Liver was exposed after sagittal incision along abdominal area. Five sections of brain and liver of approximately 0.3 in^2^ were collected and immediately frozen in liquid nitrogen and kept at −80 °C until further analysis.

### 4.3. Targeted Metabolomic Analysis

Frozen tissue samples were sent to West Coast Metabolomic Center (WCMC) (UC Davis, CA, USA) for targeted metabolomic analysis. The frozen hippocampus, medial pre-frontal cortex and liver 20 mg-aliquots were weighed and placed in 2 mL tubes. For each mg of tissue, 6 µL of methanol was added. Samples were grinded with two 3 mm stainless steel beads at 1500 rpm with Geno/Grinder (SPEX SamplePrep Metuchen, NJ, USA) for 1 min and centrifuged at 12,210 rpm for 2 min before collecting supernatant. Sample preparation was performed following protocol for the Absolute IDQ-p400 kit (Biocrates Life Sciences AG, Innsbruck, Austria), a commercially available assay which identifies 408 endogenous metabolites from eight metabolite classes including phosphatidylcholines (*n* = 172), lyso-phosphatidylcholines (*n* = 24), sphingomyelins (*n* = 31), ceramides (*n* = 9), diglycerides (*n* = 18), triglycerides (*n* = 42), acyl-carnitines (*n* = 55), amino acids (*n* = 21), biogenic amines (*n* = 21), cholesteryl esters (*n* = 14) and monosaccharides (*n* = 1). The liquid chromatography–mass spectrometry (LC-MS/MS) was used to quantify amino acids and biogenic amines while acyl-carnitines, cholesterol esters, glycerophospholipids, glycerides, sphingolipids and hexoses were assessed using Flow Injection Analysis (FIA)-(LC-MS/MS). Briefly, samples were prepared using the 96-well kit plate, adding 10 uL of the p400 internal standard mix and 10 µL of the sample supernatant and dried for 30 min under nitrogen flow and subsequently derivatized by the addition of 10 uL of a 5% solution of phenyl-isothiocyanate followed by incubation at room temperature for 20 min. The samples were then dried under nitrogen flow for 60 min and extracted by the addition of 300 µL 5 mM ammonium acetate in methanol and shaking at 450 rpm for 30 min. The extracts were collected by centrifugation into provided collection plate. For LC-MS/MS analysis, 150 µL of the extract volume were transferred and diluted 1:2 with water on an empty plate, and for the Flow Injection Analysis (FIA), the remaining 150 µL was diluted with 250 µL of the FIA mobile phase provided by the kit and added directly to the samples on the collection plate. The instrumental analysis was performed on a Thermo Vanquish UPLC coupled with a Thermo-Q Exactive hybrid quadrupole Orbitrap mass spectrometer (Thermo Scientific, Santa Clara, CA, USA) according to the guidelines from the manufacturer. The chromatography was done using the UHPLC column provided with the kit. Mobile phase A was 0.1% formic acid in H_2_O and mobile phase B 0.1% formic acid in acetonitrile. After the peaks and calibration curves were confirmed in Thermo Xcalibur, the data were preprocessed using the MetIDQ software (Biocrates, Life Science AG, Innsbruck, Austria) prior to statistical evaluation. The final concentration of metabolites (pmol/mg tissue) were generated using calibration curves for amino acids, biogenic amines and one-point calibration for 10 acyl-carnitines and sugars with the remaining metabolites within sphingolipids, glycerides, glycerophospholipids, cholesterol esters class determined by relative quantification. 

### 4.4. Statistical Analysis of Metabolomic Data

Missing values were replaced with minimum detectable values for each compound and compounds that had constant values within an organ were removed from analysis. Pretreatment of data included removal of metabolites below the limit of detection (LOD) individually defined for each metabolite as three times the background noise level. The data were analyzed using the statistical software R version 4.0.2. Principal component analysis was conducted to identify any potential outliers. To identify compounds that changed in response to treatment, the Mann–Whitney U test was performed to compare the mean rank of groups. Benjamini-Hochberg FDR correction was applied for each comparison using a threshold of FDR < 0.1. Fold changes (FC) for each compound were calculated as the median average of compound in treatment group divided by the median average of the control group. Heatmaps were generated to visualize metabolite abundance in organs from pigs fed WBM-supplemented diets with three and six servings against pigs fed the control diet. Partial least squares discriminant analyses were conducted on each organ and the variable importance in projection (vip) scores for compounds from the PLSDA was also calculated with vip values > 1.0 being indicative of the compound being important for distinguishing metabolite distribution between treatment groups [72]. The area under the curve (AUC) of the receiver operating characteristic (ROC) curve was measured for each group at each organ to determine the likelihood of a sample being correctly classified. In addition, Chemical Similarity Enrichment analysis for Metabolites (ChemRICH) was performed to calculate metabolite cluster statistics by employing the Kolmogorov–Smirnov test. A *p*-value < 0.05 indicates a statistically significant enriched compound cluster. These clusters were visualized by bubble plots, where the size of the bubble was proportional to the number of metabolites in the corresponding cluster and the color indicates the percentage of increased (red) or decreased (blue) metabolites [73].

### 4.5. RNA Sequencing and Mapping

One piece of previously frozen tissue derived from brain was homogenized with 2 mL of Qiazol (catalog 79306, Qiagen, Gaithersburg, MD, USA) using polytron homogenizer for 1 min. The homogenized sample was equally divided in two Eppendorf tubes. To each tube, 0.2 mL of chloroform was added, shaken vigorously for 30 s and incubated for 3 min at room temperature. The aqueous layer was collected after a 15 min centrifugation 2000 rpm at 4 °C and an equal volume (0.5 mL) of 70% ethanol was added before mixing and transfer to the column provided in the Qiagen RNeasy Lipid tissue mini kit (Qiagen). After several washes the RNA was eluted with 40 µL of RNase free water. With the addition of RNAseOUT (Invitrogen, Carlsbad, CA, USA) to minimize RNA degradation, residual DNA was removed using TURBO DNA-free kit (Invitrogen, Carlsbad, CA, USA). The RNA quality and concentration were assessed using an Experion RNA StdSens Analysis kit (Bio-Rad, Hercules, CA, USA). Illumina TruSeq RNA Sample Prep v2 kit (Ilumina, San Diego, CA, USA) was used to prepare the RNA samples for sequencing. RNA inputs of 1000 ng originating from individual pig samples were sequenced and gene counts were analyzed for statistical similarity among treatment groups. Sequencing library preparation involved purifying poly-A containing mRNA using magnetic beads, fragmenting the molecules, and converting them into cDNA. The cDNA was then subject to end repair, 3′-end adenylation, ligation of Illumina indexing adapters, and finally further purification and enrichment. Libraries were validated for average fragment size on the Experion Automated Electrophoresis Station using DNA 1 K chips. Library concentration was also determined using the Experion platform by implementing manual peak integration within the software.Three libraries were prepared from each pig from samples collected from medial pre-frontal cortex, hypothalamus, and hippocampus. Libraries were brought to equimolar concentrations (3–5 pM) before being combined into two 16-plex pools with a 10 pM final concentration. The sample pools were denatured and loaded onto an Illumina NextSeq 500 sequencer (San Diego, CA, USA), using two high output flow-cells per tissue to generate 150 cycle single-end reads. Base-call conversion, de-multiplexing and adapter trimming of the pooled sequence data was performed using bcl2fastq2 conversion software (v2.20.0.422, Illumina, Inc, San Diego, CA). Unaligned FASTQ files generated from sequencing were imported into CLC Bio’s Genomics Workbench (Aarhus, Denmark) where they were trimmed to remove low quality reads and adapter sequences before alignment to the swine genome Ensembl build 11.1 98_111 [27]. Because of duplications, mis-assembly and mis-annotations with this genome assembly the reads were also mapped to 12,229 unique gene sequences from of a non-redundant porcine gene library [28]. Mapped reads for each sample were summarized into gene level expression counts that were used as input for expression analysis.

### 4.6. RNA-Seq Data Analysis

The R/Bioconductor package DESeq2 (v1.28.1) was used to detect differential gene expression (DGE) between WBM-treated and control groups. Briefly, raw counts were used to estimate size factors for library size normalization. Next, gene-wise dispersions were estimated and then shrunk toward the overall dispersion trend across all genes to obtain final dispersion estimates. The negative binomial model was then fit to the raw counts and hypothesis testing was performed using the Wald test. Result statistics include the log2 fold-change (effect size), the wald test *p*-value, and the multiple testing adjusted *p*-value (Benjamini-Hochberg). Genes were considered differentially expressed if their adjusted *p*-value was <0.05 [74,75].

### 4.7. Differentially Expressed Genes-Real Time PCR Validation

Real time PCR analysis was used to validate selected significantly changed DEGs associated with dietary treatment that were identified by RNA-seq analysis using RNA converted to cDNA via the iScript cDNA synthesis Kit (Biorad, Hercules, CA, USA). Briefly, 25 ng/well of cDNA was used for real time PCR amplification using Powerup SYBR-green DNA Polymerase master mix (Life Technologies, Carlsbad, CA, USA) and the ABI PRISM 7500 Sequence detector system (Applied Biosystems, Foster City, CA, USA). Amplification conditions were: 50 °C for 2 min; 95 °C for 2 min; 40 cycles of 95 °C for 15 s and 60 °C for 1 min with a three-step dissociation curve with 95 °C for 15 s, 60 °C for 1 min, and 95 °C for 15 s. Specific porcine primers and probe sequences against selected genes were synthesized by Biosearch Technologies (Novato, CA, USA) as described in the Porcine Translational Research Database [28]. Gene expression was normalized to the housekeeping gene RPL32 using the 2^−ΔΔ^CT method [76].

### 4.8. 16 S rDNA Amplicon Multi-Tag Sequencing Data and Metabolite Correlations

Bacterial taxa abundance differences in all treatment groups previously defined by Quantitative Insights into Microbial Ecology (QIIME2) analysis [26] were used to construct a Correlation Network with tissue metabolites generated in this study using a Pearson correlation between all features in each sample class at *p*-value of 0.05. Significant correlations (*p* < 0.05) between metabolites and microbial taxa were summarized in heatmaps. A correlation difference between treatments for each tissue between times was also calculated and visualized using Cytoscape v3.9.0, an open source software platform used for visualization of interactive complex networks and biological pathways [77].

## Figures and Tables

**Table 1:** Targeted Metabolomic compound distribution among metabolite classes.

Metabolite Class	Liver	Cortex	Hippocampus
Acyl-carnitines (55) *	3	3	3
Amino acids (21)	17	17	17
Biogenic Amines (21)	11	7	7
Cholesterol Esters (14)	5	5	5
Diglycerides (18)	6	2	2
Triglycerides (42)	1	1	1
Phosphatidylcholine (172)	92	93	96
Lyso-phosphatidylcholine (24)	8	8	8
Sphingomyelins (31)	21	16	17
Ceramides (9)	2	1	1
Monosaccharides (1)	1	1	1
total (408)	167	154	158

* Number in parentheses indicate the number of available metabolites per Class.

**Table 2:** Brain Cortex metabolites differences among treatment groups.

		Mean Rank			3 Serv. vs. Control		6 Serv. vs. Control		6 Serv. vs. 3 Serv.		Cortex Vip
Metabolite	Class	Control	3 Servings	6 Servings	FC	FDR	FC	FDR	FC	FDR	
Arg	Aminoacids	13.11 *	10.33	18.56	0.94	0.340	1.20	0.141	1.27	0.141	1.93
Gln	Aminoacids	10.56	13.33	18.11	1.12	0.730	1.16	0.056	1.03	0.581	1.54
Met	Aminoacids	11.11	12.33	18.56	0.99	1.000	1.23	0.073	1.24	0.285	1.47
Thr	Aminoacids	8.61	15.50	17.89	1.11	0.183	1.35	**0.017**	1.22	0.730	1.41
Taurine	Biogenic Amines	12.56	10.72	18.72	0.86	0.596	1.12	0.140	1.31	0.140	1.58
DG (42:2)	Glycerides	9.78	13.06	19.17	1.07	0.401	1.18	**0.045**	1.10	0.170	2.05
PC (29:1)	Glycerophospholipids	10.72	12.83	18.44	1.10	0.562	1.22	0.154	1.11	0.200	1.80
LPC (20:4)	Glycerophospholipids	10.17	13.28	18.56	1.04	0.401	1.13	0.102	1.09	0.242	1.74
PC-O (40:4)	Glycerophospholipids	10.89	13.17	17.94	1.61	0.436	1.86	0.217	1.15	0.217	1.74
PC-O (34:3)	Glycerophospholipids	11.11	12.78	18.11	1.17	0.507	1.38	0.183	1.18	0.183	1.64
PC-O (38:5)	Glycerophospholipids	13.22	11.44	17.33	0.92	0.605	1.15	0.387	1.25	0.387	1.57
PC-O (40:5)	Glycerophospholipids	12.00	12.11	17.89	1.07	1.000	1.13	0.200	1.06	0.200	1.49
PC (33:5)	Glycerophospholipids	10.11	13.94	17.94	1.07	0.331	1.14	0.125	1.06	0.331	1.46
PC (34:2)	Glycerophospholipids	11.17	13.06	17.78	0.99	0.627	1.08	0.278	1.09	0.324	1.46
PC (31:1)	Glycerophospholipids	12.72	11.33	17.94	0.99	0.626	1.04	0.200	1.05	0.200	1.45
SM (38:2)	Sphingolipids	11.11	12.22	18.67	1.02	0.796	1.18	0.141	1.15	0.141	2.14
SM (32:1)	Sphingolipids	10.11	12.89	19.00	1.03	0.387	1.49	0.094	1.44	0.115	2.12
SM (34:2)	Sphingolipids	11.89	11.56	18.56	1.08	0.931	1.28	0.115	1.19	0.115	1.54

* Values represent the Mann–Whitney mean rank of treatment groups. Fold change values for each comparison were calculated with *p*-values corrected for false discovery rate—Benjamini–Hochberg method (FDR) for each Mann–Whitney U test. The variable importance projection score from Partial least squares discriminant analysis was also calculated, Values > 1 are indicative of the compound being important for distinguishing between groups. FDR < 0.05 are in shown in bold.

**Table 3:** Brain Hippocampus metabolites differences among groups.

-	-	Mean Ranks			3 Serv. vs. Control		6 Serv. vs. Control		6 Serv. vs. 3 Serv.		Hippocampus VIP
Metabolite	Class	Control	3 Serv.	6 Serv.	FC	FDR	FC	FDR	FC	FDR	
Taurine	Biogenic Amines	12 *	11.56	18.44	0.82	0.863	1.26	0.141	1.54	0.141	1.48
CE (19:2)	Cholesterol Esters	16.17	9.56	16.28	0.85	0.153	1.06	0.931	1.24	0.153	1.86
DG (38:5)	Glycerides	14.67	9.78	17.56	0.79	0.285	1.64	0.436	2.08	0.151	1.83
PC-O (40:3)	Glycerophospholipids	11.33	10.44	20.22	0.85	0.48	2.08	**0.008**	2.46	0.063	2.24
PC (39:3)	Glycerophospholipids	14.39	10.22	17.39	0.86	0.376	1.00	0.401	1.17	0.232	1.96
PC (41:4)	Glycerophospholipids	13.56	10.78	17.67	0.78	0.436	1.25	0.387	1.60	0.282	1.80
PC (39:4)	Glycerophospholipids	14.89	10.83	16.28	0.89	0.465	1.05	0.757	1.17	0.465	1.67
PC (33:2)	Glycerophospholipids	11.94	11.89	18.17	1.03	1.000	1.51	0.183	1.47	0.183	1.66
PC (42:4)	Glycerophospholipids	17.22	10.17	14.61	0.83	0.19	0.97	0.536	1.17	0.404	1.65
PC (36:3)	Glycerophospholipids	14.67	10.56	16.78	0.80	0.426	0.99	0.594	1.24	0.333	1.61
PC (37:1)	Glycerophospholipids	13.22	11.61	17.17	0.51	0.666	1.27	0.434	2.51	0.434	1.61
PC (38:2)	Glycerophospholipids	14.44	10.22	17.33	0.74	0.35	1.05	0.401	1.41	0.255	1.52
PC (37:2)	Glycerophospholipids	14.33	10.89	16.78	0.76	0.436	1.17	0.436	1.54	0.436	1.50
PC (36:1)	Glycerophospholipids	13.94	11.22	16.83	0.80	0.387	1.07	0.387	1.34	0.387	1.49
PC (39:5)	Glycerophospholipids	20.72	9.72	11.56	0.84	**0.009**	0.88	**0.032**	1.05	0.723	1.35
PC (42:6)	Glycerophospholipids	19.89	11.33	10.78	0.82	**0.024**	0.75	0.077	0.92	0.595	1.00
PC-O (33:3)	Glycerophospholipids	13.22	11.00	17.78	0.87	0.436	1.58	0.242	1.83	0.242	1.48
PC (31:1)	Glycerophospholipids	12.61	12.67	16.72	0.99	1.000	1.06	0.510	1.07	0.510	1.46
PC-O (33:4)	Glycerophospholipids	13.22	11.44	17.33	0.85	0.596	1.13	0.387	1.34	0.387	1.45
SM (38:2)	Sphingolipids	13.11	11.00	17.89	0.99	0.536	1.33	0.277	1.34	0.277	1.87
SM (41:2)	Sphingolipids	12.44	11.28	18.28	0.74	0.791	1.38	0.200	1.87	0.200	1.81
SM (40:2)	Sphingolipids	12.11	11.39	18.50	0.92	0.73	1.58	0.153	1.71	0.153	1.74
SM (43:2)	Sphingolipids	13.00	11.22	17.78	0.95	0.605	1.44	0.285	1.51	0.285	1.69
SM (42:2)	Sphingolipids	13.00	10.89	18.11	0.84	0.536	1.48	0.236	1.77	0.231	1.68

* Values represent the Mann–Whitney mean rank of treatment groups. Fold change values for each comparison were calculated with *p*-values corrected for false discovery rate—Benjamini–Hochberg method (FDR) for each Mann–Whitney U test. The variable importance projection score from Partial least squares discriminant analysis was also calculated, Values > 1 are indicative of the compound being important for distinguishing between groups. FDR < 0.05 are in shown in bold.

**Table 4:** Liver metabolite differences among treatment groups.

-	-	Mean Rank			3 Serv. vs. Control		6 Serv. vs. Control		6 Serv. vs. 3 Serv.		Liver VIP-
Metabolite	Class	Control	3 Serv.	6 Serv.	FC	FDR	FC	FDR	FC	FDR	
AC (4:0-OH)	Acylcarnitines	10.25 *	16.17	13.72	1.12	0.275	1.13	0.627	1.01	0.627	1.54
Serotonin	Biogenic Amines	9.19	20.89	9.94	2.40	**0.007**	1.09	0.700	0.45	**0.001**	2.07
CE (22:5)	Cholesterol Esters	17.63	11.44	11.89	0.86	0.204	0.86	0.204	1.00	0.930	1.49
DG (36:2)	Glycerides	18.00	9.61	13.39	0.60	0.162	0.88	0.222	1.48	0.222	1.67
PC (31:1)	Glycerophospholipids	20.00	9.33	11.89	0.61	**0.024**	0.62	**0.031**	1.03	0.427	2.31
PC (35:1)	Glycerophospholipids	19.31	9.39	12.44	0.64	**0.017**	0.82	0.125	1.28	0.453	2.26
PC-O (33:4)	Glycerophospholipids	19.38	8.56	13.22	0.73	**0.017**	0.77	0.112	1.05	0.161	2.23
PC-O (35:4)	Glycerophospholipids	19.69	9.78	11.72	0.46	**0.029**	0.62	**0.047**	1.33	0.574	2.18
PC (46:2)	Glycerophospholipids	19.63	9.78	11.78	0.39	**0.034**	0.47	**0.034**	1.19	0.418	2.16
LPC (15:0)	Glycerophospholipids	19.25	10.67	11.22	0.67	**0.045**	0.83	**0.045**	1.23	0.860	2.14
PC (39:3)	Glycerophospholipids	18.63	8.00	14.44	0.72	**0.024**	0.85	0.228	1.18	0.095	2.12
PC (33:1)	Glycerophospholipids	18.88	9.44	12.78	0.66	**0.024**	0.74	0.209	1.12	0.436	2.07
PC (35:4)	Glycerophospholipids	20.50	10.00	10.78	0.86	**0.014**	0.85	**0.021**	0.99	0.930	1.98
PC-O (33:3)	Glycerophospholipids	17.88	10.11	13.00	0.72	0.139	0.75	0.300	1.04	0.436	1.91
PC (37:1)	Glycerophospholipids	19.00	10.67	11.44	0.75	0.070	0.84	0.070	1.11	0.863	1.86
PC (32:6)	Glycerophospholipids	7.19	15.89	16.72	1.61	**0.027**	1.44	**0.027**	0.89	0.791	1.84
PC (38:2)	Glycerophospholipids	8.00	17.72	14.17	2.00	0.037	1.86	0.152	0.93	0.331	1.81
PC (31:0)	Glycerophospholipids	17.50	11.78	11.67	0.97	0.290	0.87	0.275	0.89	0.931	1.65
PC (33:0)	Glycerophospholipids	17.63	10.28	13.06	0.79	0.178	0.92	0.354	1.17	0.453	1.64
PC (30:0)	Glycerophospholipids	17.88	11.33	11.78	0.84	0.171	0.76	0.171	0.90	0.931	1.60
PC (32:1)	Glycerophospholipids	17.81	10.39	12.78	0.77	0.103	0.89	0.402	1.16	0.659	1.48
SM (43:1)	Sphingolipids	20.50	9.67	11.11	0.61	**0.008**	0.64	**0.008**	1.06	0.605	2.43
SM (42:1)	Sphingolipids	20.38	11.17	9.72	0.72	**0.018**	0.63	**0.018**	0.88	0.666	2.16
Cer (42:1)	Sphingolipids	20.13	8.78	12.33	0.61	**0.007**	0.78	**0.041**	1.28	0.297	2.16
SM (41:1)	Sphingolipids	17.88	11.78	11.33	0.92	0.171	0.86	0.171	0.93	0.931	1.81
Cer (42:2)	Sphingolipids	17.63	9.89	13.44	0.65	0.223	0.75	0.258	1.17	0.258	1.58

* Values represent the Mann–Whitney mean rank of treatment groups. Fold change values for each comparison were calculated with *p*-values corrected for false discovery rate—Benjamini–Hochberg method (FDR) for each Mann–Whitney U test. The variable importance projection score from Partial least squares discriminant analysis was also calculated, Values > 1 are indicative of the compound being important for distinguishing between groups. FDR < 0.05 are in shown in bold.

## Data Availability

The data presented in this study are available in article or Supplementary Materials.

## Supplementary Materials

The following are available online at https://www.mdpi.com/article/10.3390/metabo11110779/s1, Figure S1: Comparisons of Univariate analysis between groups, Figure S2: Correlation between bacteria in the fecal microbiome and several metabolites in the cortex of pigs fed six-servings of a WBM-supplemented diet, Figure S3: Correlation of bacteria in the fecal microbiome and metabolites in the liver from pigs fed six servings of a WBM-supplemented diet, Figure S4: Real time PCR of mitochondrial gene *mtATP8* in the brain cortex, Table S1: Microbiota-metabolite network plots in Hippocampus, Cortex and Liver of pigs fed 6 serv and control diets.
