# Supplementary material for: The Effects of Consuming White Button Mushroom Agaricus bisporus on the Brain and Liver Metabolome Using a Targeted Metabolomic Analysis
Source: Metabolites. 2021 Nov 15;11(11):779. doi: 10.3390/metabo11110779 (PMC8625434; doi:10.3390/metabo11110779)
Supplement: Supplementary file 1 [file metabolites-11-00779-s001.zip › metabolites-1410532-supplementary.pdf]

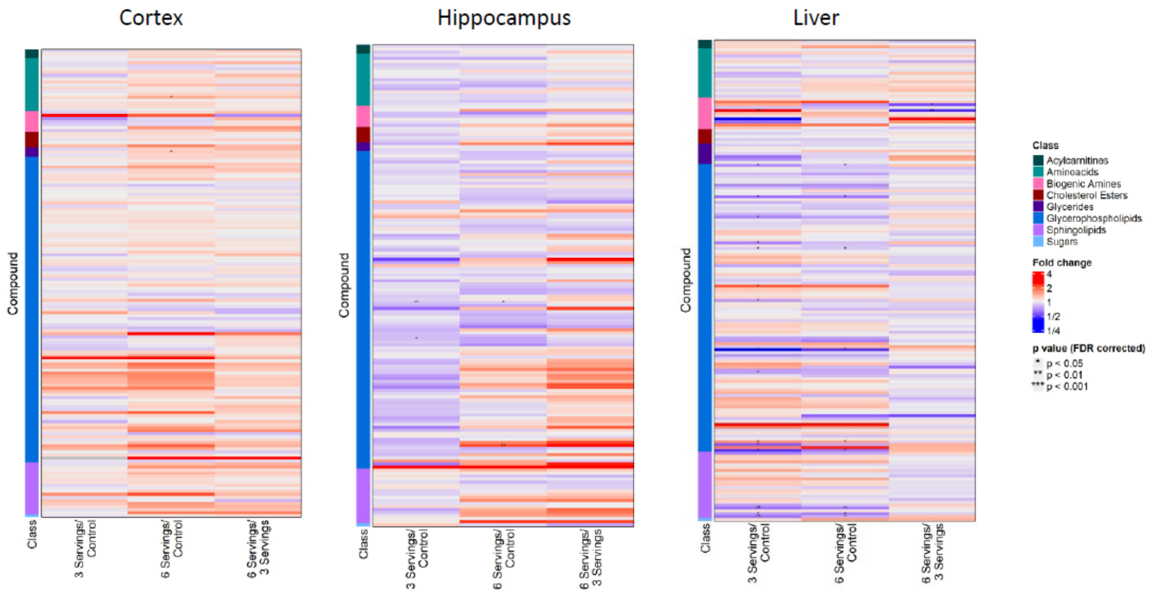

Supplementary Figure S1. Comparisons of Univariate analysis between groups. Three heatmaps were created to show the results of the univariate analysis. For each of the three organs, a heatmap with corrected p values were created. Each row is a compound, and each column is a pairwise comparison. The color represents the fold change value (red indicates that the metabolites is more abundant in group 1 than in group 2, and blue indicates the opposite. Asterisks indicate p-values (one asterisk indicates p<0.05; two asterisks indicate p<0.01). Metabolite class information is represented by the color displayed in the legend bar.

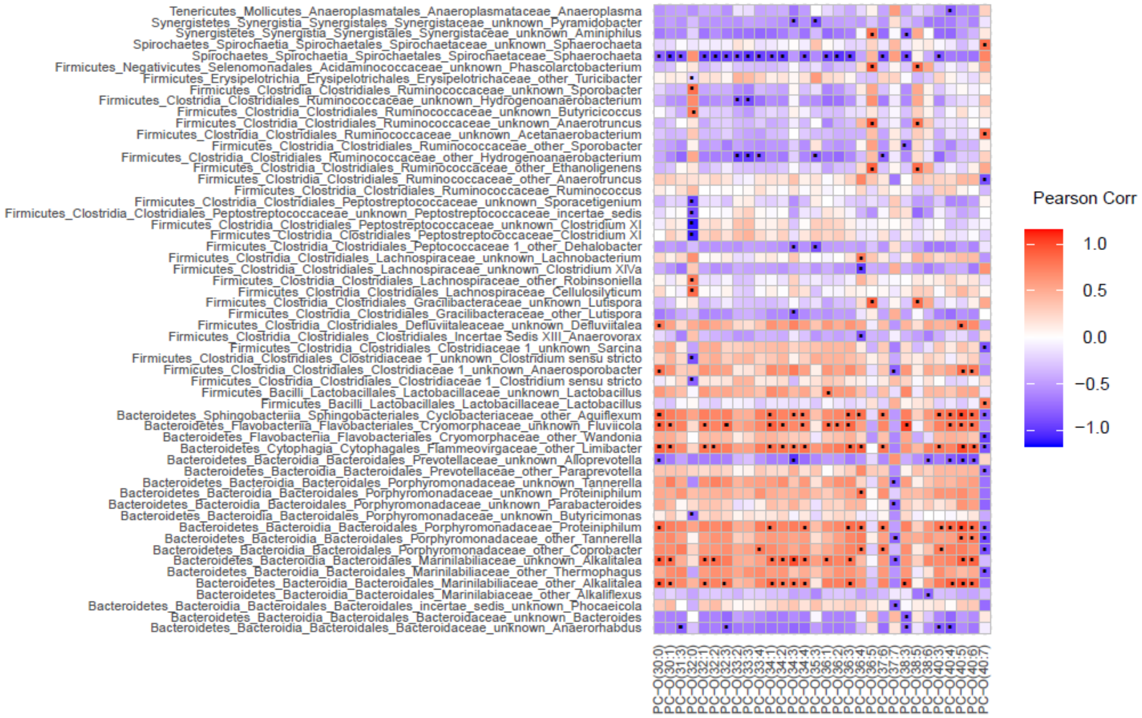

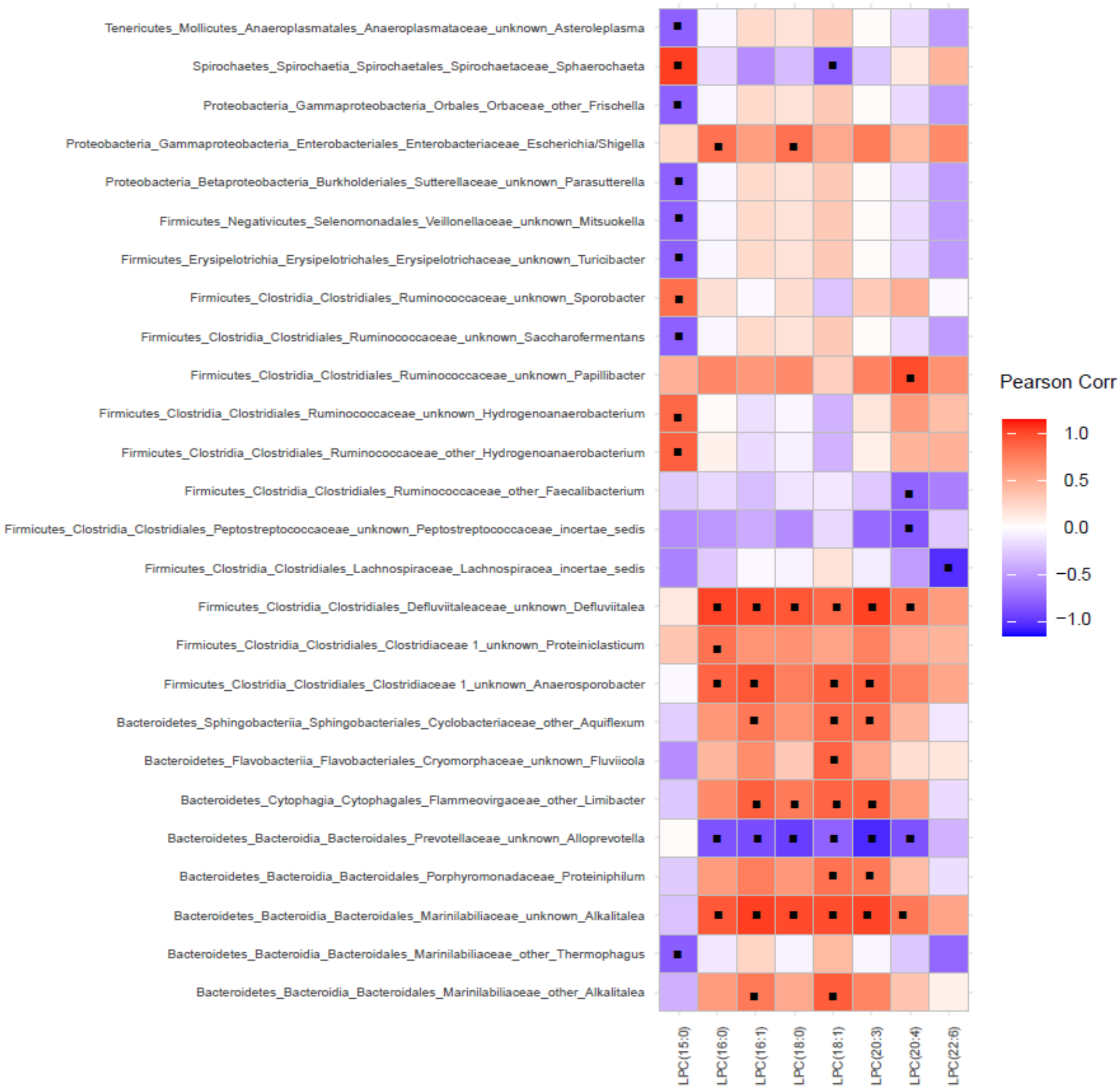

Tenericutes_Mollicutes_Anaeroplasmatales_Anaeroplasmataceae_unknown_Asteroleplasma
Spirochaetes_Spirochaetia_Spirochaetales_Spirochaetaceae_Sphaerochaeta
Proteobacteria_Gammaproteobacteria_Orbales_Orbaceae_other_Frischella
Proteobacteria_Gammaproteobacteria_Enterobacteriales_Enterobacteriaceae_Escherichia/Shigella
Proteobacteria_Betaproteobacteria_Burkholderiales_Sutterellaceae_unknown_Parasutterella
Firmicutes_Negativicutes_Selenomonadales_Veillonellaceae_unknown_Mitsuokella
Firmicutes_Erysipelotrichia_Erysipelotrichales_Erysipelotrichaceae_unknown_Turicibacter
Firmicutes_Clostridia_Clostridiales_Ruminococcaceae_unknown_Sporobacter
Firmicutes_Clostridia_Clostridiales_Ruminococcaceae_unknown_Saccharofermentans
Firmicutes_Clostridia_Clostridiales_Ruminococcaceae_unknown_Papillibacter
Firmicutes_Clostridia_Clostridiales_Ruminococcaceae_unknown_Hydrogenoanaerobacterium
Firmicutes_Clostridia_Clostridiales_Ruminococcaceae_other_Hydrogenoanaerobacterium
Firmicutes_Clostridia_Clostridiales_Ruminococcaceae_other_Faecalibacterium
Firmicutes_Clostridia_Clostridiales_Peptostreptococcaceae_unknown_Peptostreptococcaceae_incertae_sedis
Firmicutes_Clostridia_Clostridiales_Lachnospiraceae_Lachnospiracea_incertae_sedis
Firmicutes_Clostridia_Clostridiales_Defluviitaleaceae_unknown_Defluviitalea
Firmicutes_Clostridia_Clostridiales_Clostridiaceae 1_unknown_Proteiniclasticum
Firmicutes_Clostridia_Clostridiales_Clostridiaceae 1_unknown_Anaerosporobacter
Bacteroidetes_Sphingobacteriia_Sphingobacteriales_Cyclobacteriaceae_other_Aquiflexum
Bacteroidetes_Flavobacteriia_Flavobacteriales_Cryomorphaceae_unknown_Fluviicola
Bacteroidetes_Cytophagia_Cytophagales_Flammeovirgaceae_other_Limibacter
Bacteroidetes_Bacteroidia_Bacteroidales_Prevotellaceae_unknown_Alloprevotella
Bacteroidetes_Bacteroidia_Bacteroidales_Porphyromonadaceae_Proteiniphilum
Bacteroidetes_Bacteroidia_Bacteroidales_Marinilabiliaceae_unknown_Alkalitalea
Bacteroidetes_Bacteroidia_Bacteroidales_Marinilabiliaceae_other_Thermophagus
Bacteroidetes_Bacteroidia_Bacteroidales_Marinilabiliaceae_other_Alkalitalea
LPC(15:0)
LPC(16:0)
LPC(16:1)
LPC(18:0)
LPC(18:1)
LPC(20:3)
LPC(20:4)
LPC(22:6)
Pearson Corr
1.0
0.5
0.0
−0.5
−1.0

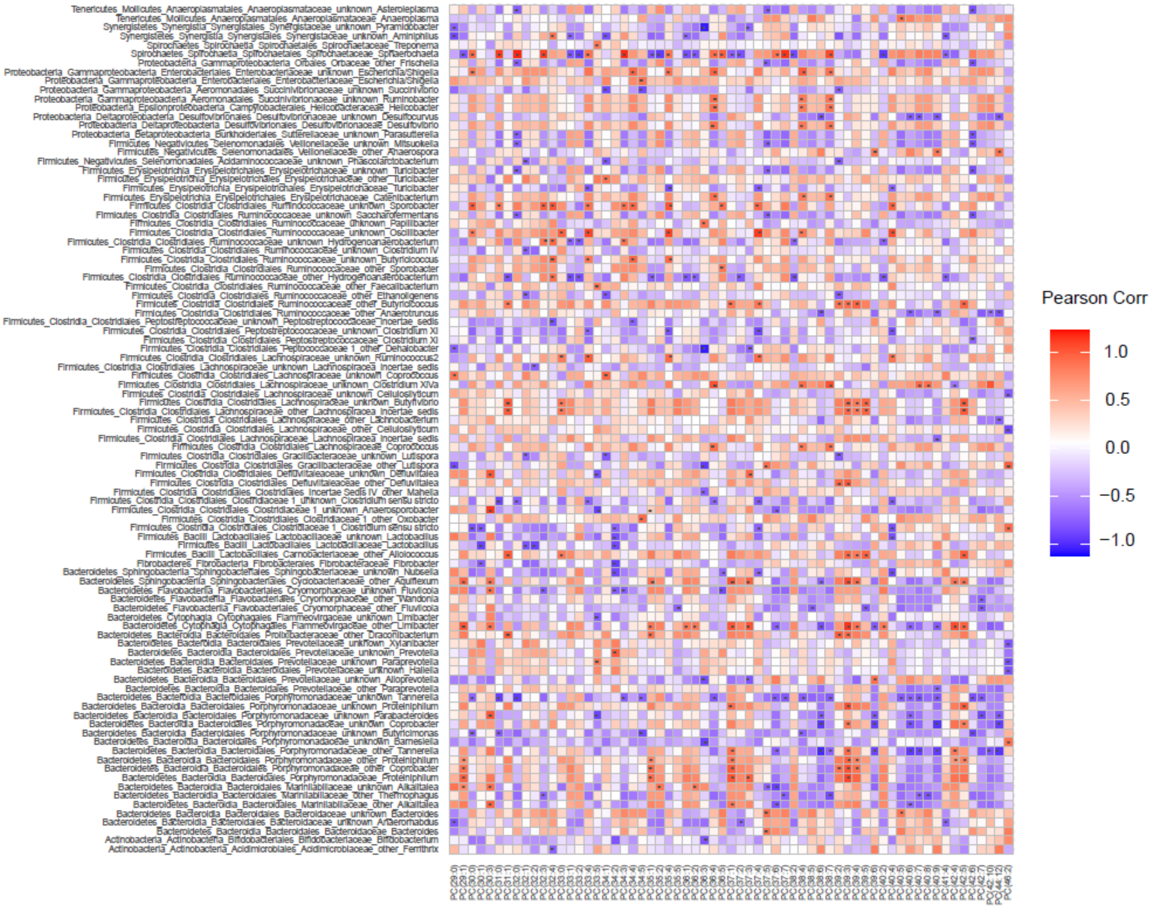
Pearson Corr
1.0
0.5
0.0
−0.5
−1.0

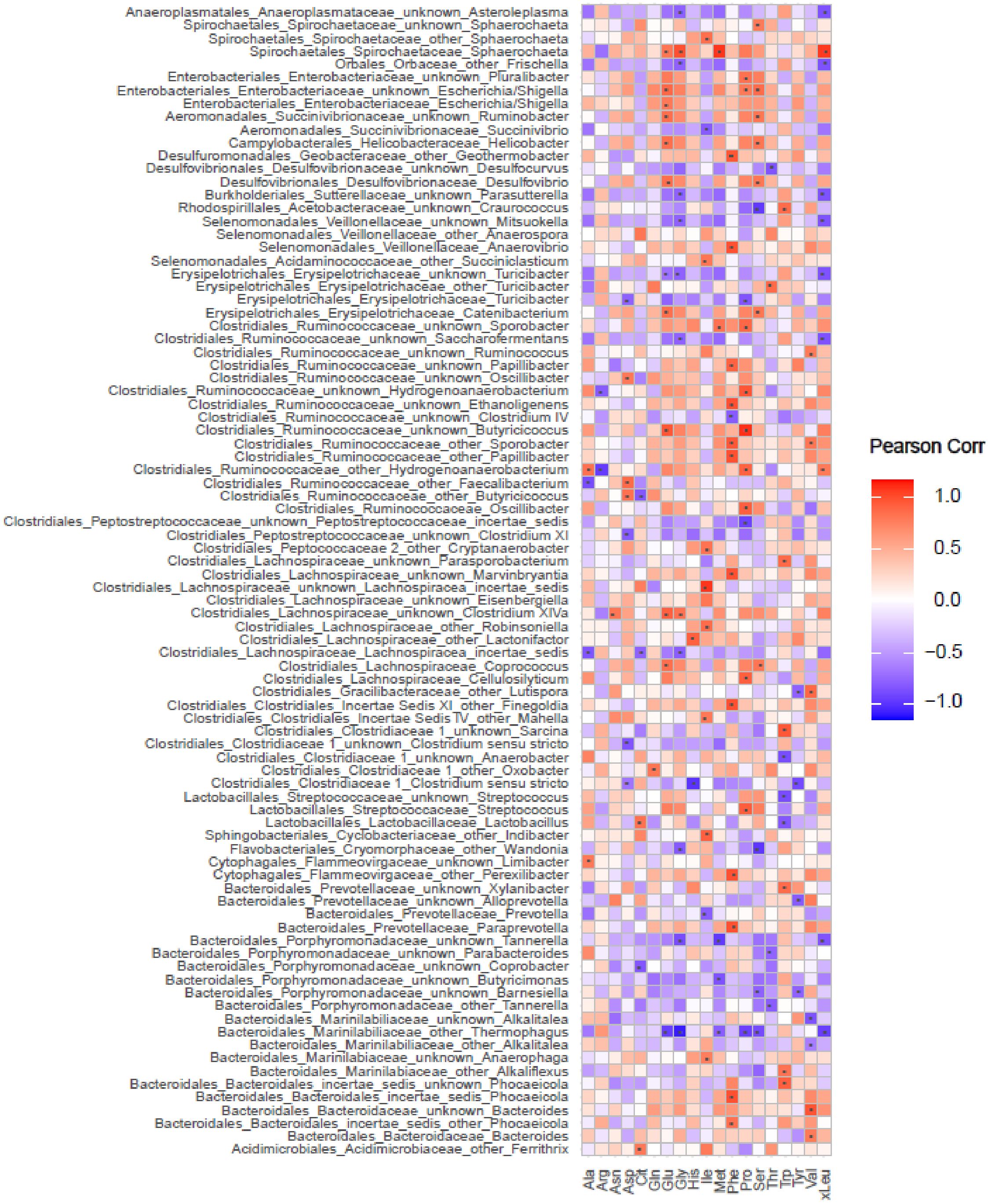

Supplementary Figure S2. Correlation between bacteria in the fecal microbiome and metabolites in the cortex of pigs fed six-servings of a WBM supplemented diet. Several lipid species within glycerophospholipids (alkyl-acyl-glycerol phosphocholines (PC-O)(S2-1), lyso-phosphatidylcholines (LPC)(S2-2) and phosphatidylcholines (PC)(S2-3) and amino acids (S2-4) in the cortex of pigs fed six servings of a WBM supplemented diet were correlated with bacteria in the fecal microbiome. Pearson Correlation coefficients were estimated for each pairwise comparison of bacterial taxa and metabolite with direction of correlation represented by color according to legend. Black dots within heatmap represent significant relationships (p value<0.05).

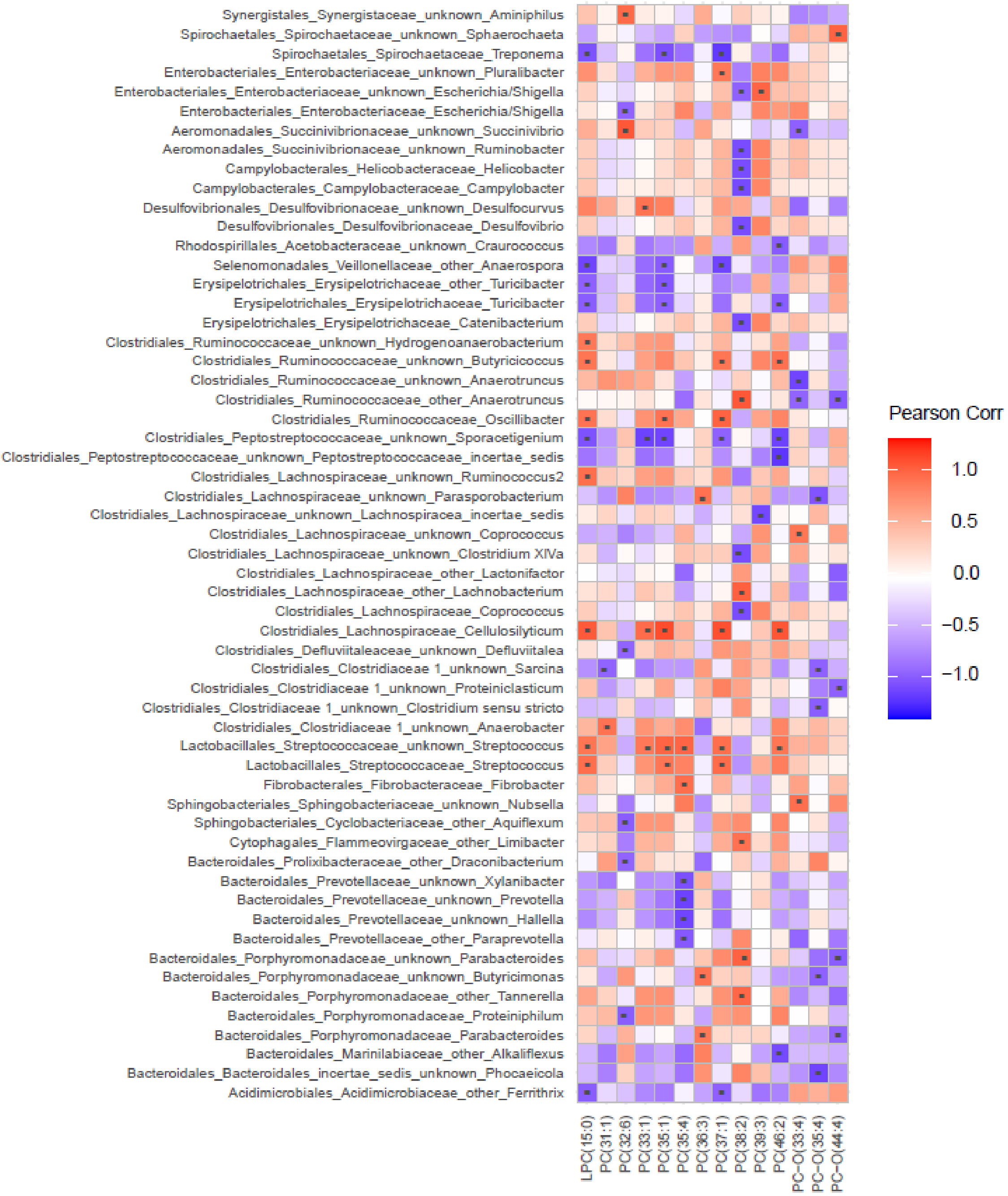
Synergistales_Synergistaceae_unknown_Aminiphilus
Spirochaetales_Spirochaetaceae_unknown_Sphaerochaeta
Spirochaetales_Spirochaetaceae_Treponema
Enterobacteriales_Enterobacteriaceae_unknown_Pluralibacter
Enterobacteriales_Enterobacteriaceae_unknown_Escherichia/Shigella
Enterobacteriales_Enterobacteriaceae_Escherichia/Shigella
Aeromonadales_Succinivibrionaceae_unknown_Succinivibrio
Aeromonadales_Succinivibrionaceae_unknown_Ruminobacter
Campylobacterales_Helicobacteraceae_Helicobacter
Campylobacterales_Campylobacteraceae_Campylobacter
Desulfovibrionales_Desulfovibrionaceae_unknown_Desulfocurvus
Desulfovibrionales_Desulfovibrionaceae_Desulfovibrio
Rhodospirillales_Acetobacteraceae_unknown_Craurococcus
Selenomonadales_Veillonellaceae_other_Anaerospora
Erysipelotrichales_Erysipelotrichaceae_other_Turicibacter
Erysipelotrichales_Erysipelotrichaceae_Turicibacter
Erysipelotrichales_Erysipelotrichaceae_Catenibacterium
Clostridiales_Ruminococcaceae_unknown_Hydrogenoanaerobacterium
Clostridiales_Ruminococcaceae_unknown_Butyricicoccus
Clostridiales_Ruminococcaceae_unknown_Anaerotruncus
Clostridiales_Ruminococcaceae_other_Anaerotruncus
Clostridiales_Ruminococcaceae_Oscillibacter
Clostridiales_Peptostreptococcaceae_unknown_Sporacetigenium
Clostridiales_Peptostreptococcaceae_unknown_Peptostreptococcaceae_incertae_sedis
Clostridiales_Lachnospiraceae_unknown_Ruminococcus2
Clostridiales_Lachnospiraceae_unknown_Parasporobacterium
Clostridiales_Lachnospiraceae_unknown_Lachnospiracea_incertae_sedis
Clostridiales_Lachnospiraceae_unknown_Coprococcus
Clostridiales_Lachnospiraceae_unknown_Clostridium XlVa
Clostridiales_Lachnospiraceae_other_Lactonifactor
Clostridiales_Lachnospiraceae_other_Lachnobacterium
Clostridiales_Lachnospiraceae_Coprococcus
Clostridiales_Lachnospiraceae_Cellulosilyticum
Clostridiales_Defluviitaleaceae_unknown_Defluviitalea
Clostridiales_Clostridiaceae 1_unknown_Sarcina
Clostridiales_Clostridiaceae 1_unknown_Proteiniclasticum
Clostridiales_Clostridiaceae 1_unknown_Clostridium sensu stricto
Clostridiales_Clostridiaceae 1_unknown_Anaerobacter
Lactobacillales_Streptococcaceae_unknown_Streptococcus
Lactobacillales_Streptococcaceae_Streptococcus
Fibrobacterales_Fibrobacteraceae_Fibrobacter
Sphingobacteriales_Sphingobacteriaceae_unknown_Nubsella
Sphingobacteriales_Cyclobacteriaceae_other_Aquiflexum
Cytophagales_Flammeovirgaceae_other_Limibacter
Bacteroidales_Prolixibacteraceae_other_Draconibacterium
Bacteroidales_Prevotellaceae_unknown_Xylanibacter
Bacteroidales_Prevotellaceae_unknown_Prevotella
Bacteroidales_Prevotellaceae_unknown_Hallella
Bacteroidales_Prevotellaceae_other_Paraprevotella
Bacteroidales_Porphyromonadaceae_unknown_Parabacteroides
Bacteroidales_Porphyromonadaceae_unknown_Butyricimonas
Bacteroidales_Porphyromonadaceae_other_Tannerella
Bacteroidales_Porphyromonadaceae_Proteiniphilum
Bacteroidales_Porphyromonadaceae_Parabacteroides
Bacteroidales_Marinilabiaceae_other_Alkaliflexus
Bacteroidales_Bacteroidales_incertae_sedis_unknown_Phocaeicola
Acidimicrobiales_Acidimicrobiaceae_other_Ferrithrix
LPC(15:0)
PC(31:1)
PC(32:6)
PC(33:1)
PC(35:1)
PC(35:4)
PC(36:3)
PC(37:1)
PC(38:2)
PC(39:3)
PC(46:2)
PC−O(33:4)
PC−O(35:4)
PC−O(44:4)
Pearson Corr
1.0
0.5
0.0
−0.5
−1.0

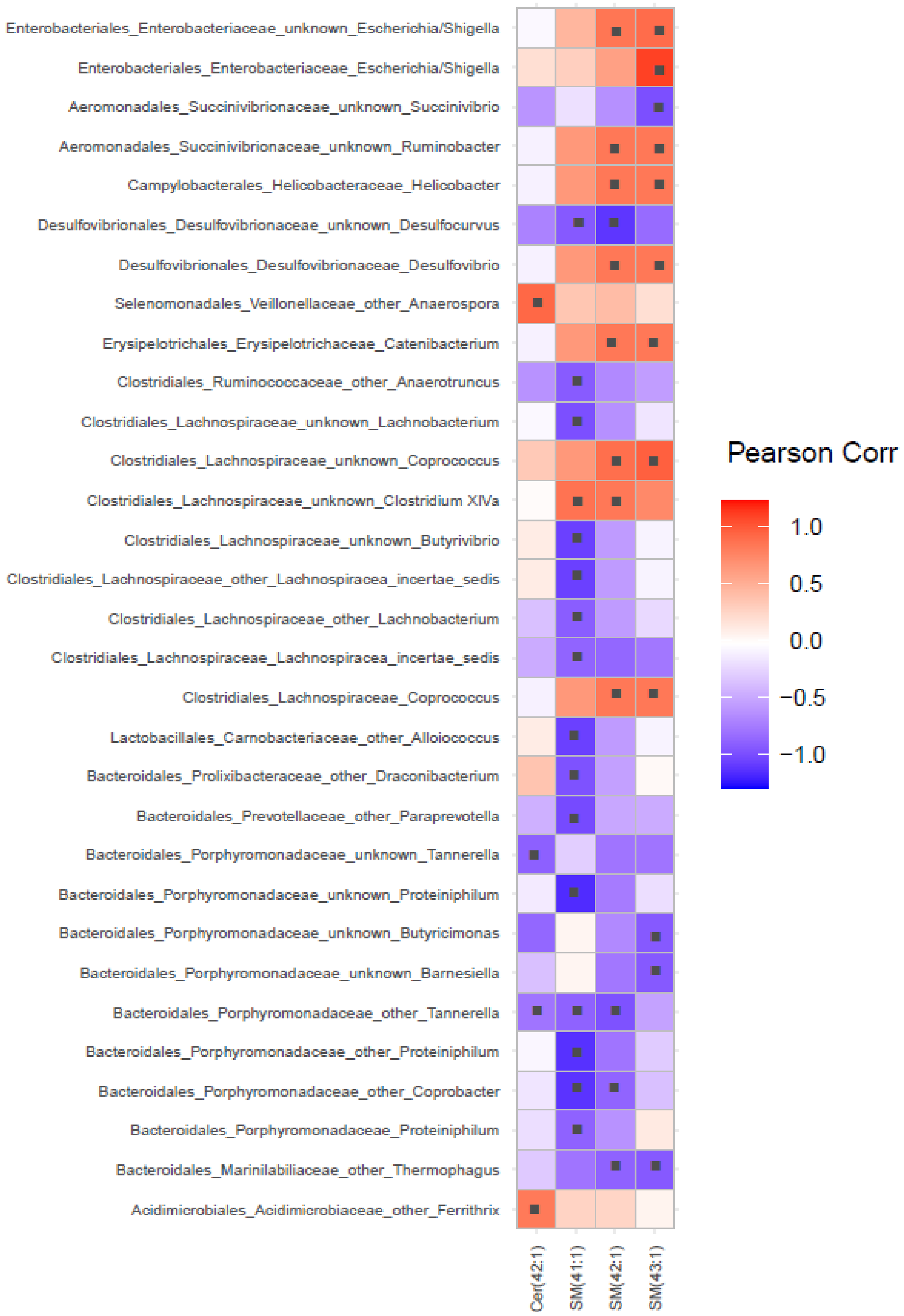

Supplementary Figure S3. Correlation of bacteria in the fecal microbiome and metabolites in the liver from pigs fed six servings of a WBM supplemented diet. Most abundant metabolites classes of phosphatidylcholine (S3-1)and sphingolipids (S3-2) were correlated with bacteria in the fecal microbiome. Pearson correlation coefficients were estimated for each pairwise comparison of bacterial taxa and metabolite with direction of correlation represented by color according to legend. Black dots within heatmap represent significant relationships (p value<0.05).

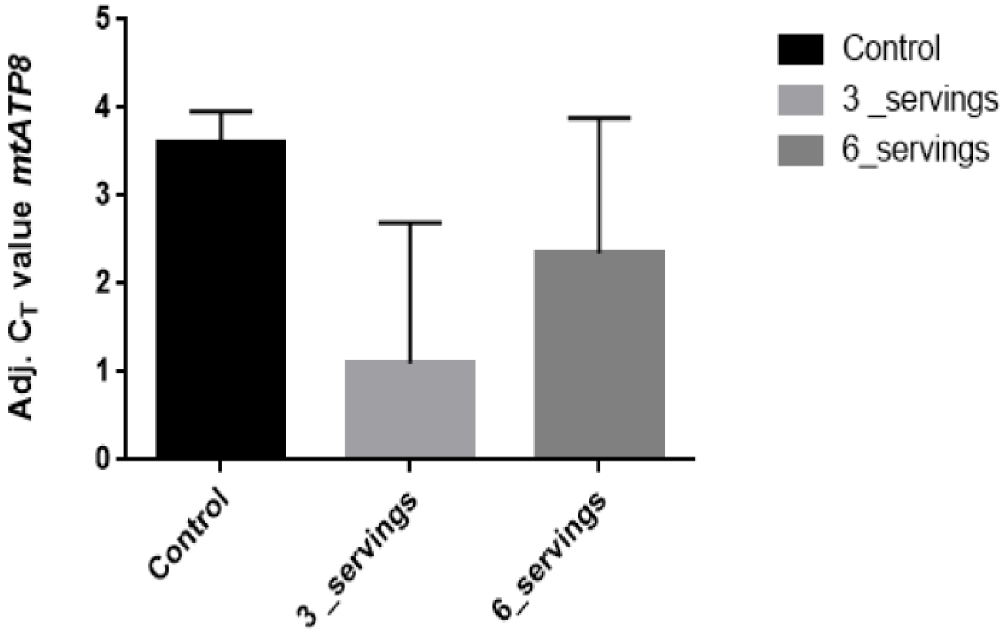

Supplementary Figure S4. Real time PCR of mitochondrial gene *mtATP8* in the brain cortex. Bars represent average adjusted Ct values for mtATP8 gene expression in the cortex of pigs fed three or six servings of WBM supplemented diets compared to a control diet.
